# Conflict and Climate Factors and the Risk of Child Acute Malnutrition Among Children Aged 24–59 Months: A Comparative Analysis of Kenya, Nigeria, and Uganda

**DOI:** 10.1007/s40980-021-00102-w

**Published:** 2022-02-01

**Authors:** Kathryn Grace, Andrew Verdin, Molly Brown, Maryia Bakhtsiyarava, David Backer, Trey Billing

**Affiliations:** 1 University of Minnesota, Minneapolis, USA; 2 University of Maryland, College Park, USA; 3 The Ohio State University, Ohio, USA; 4 University of California at Berkeley, Berkeley, USA

**Keywords:** Armed conflict, Food insecurity, Climate, Sub-Saharan Africa, Acute malnutrition

## Abstract

Acute malnutrition affects a sizeable number of young children around the world, with serious repercussions for mortality and morbidity. Among the top priorities in addressing this problem are to anticipate which children tend to be susceptible and where and when crises of high prevalence rates would be likely to arise. In this article, we highlight the potential role of conflict and climate conditions as risk factors for acute malnutrition, while also assessing other vulnerabilities at the individual- and household-levels. Existing research reflects these features selectively, whereas we incorporate all the features into the same study. The empirical analysis relies on integration of health, conflict, and environmental data at multiple scales of observation to focuses on how local conflict and climate factors relate to an individual child’s health. The centerpiece of the analysis is data from the Demographic and Health Surveys conducted in several different cross-sectional waves covering 2003–2016 in Kenya, Nigeria, and Uganda. The results obtained from multi-level statistical models indicate that in Kenya and Nigeria, conflict is associated with lower weight-for-height scores among children, even after accounting for individual-level and climate factors. In Nigeria and Kenya, conflict lagged 1–3 months and occurring within the growing season tends to reduce WHZ scores. In Uganda, however, weight-for-height scores are primarily associated with individual-level and household-level conditions and demonstrate little association with conflict or climate factors. The findings are valuable to guide humanitarian policymakers and practitioners in effective and efficient targeting of attention, interventions, and resources that lessen burdens of acute malnutrition in countries prone to conflict and climate shocks.

## 1 Introduction

Worldwide, nearly half of the deaths of children below 5 years of age result from malnutrition (WHO, 2020). Of particular concern is acute malnutrition, characterized by a sudden, rapid decrease in caloric intake resulting in a reduced weight-for-height z-score (WHZ) or in wasting (WHZ < −2) (UNICEF/WHO/World Bank, 2019). While low WHZ is broadly associated with food insecurity and insufficient caloric and nutritional intake, research has shown that conflict and climate conditions may increase the risk of malnutrition by acting through factors (e.g., reduced agricultural production, reduced access to markets and health/humanitarian aid) more directly related to food security (Delbiso et al., 2017, Thiede & Stube, 2020, Phalkey et al., 2015, Baker & Anttila-Hughes, 2020, Martin-Shields & Stojetz, 2019). Researchers generally evaluate how food security and malnutrition are affected by conflict conditions (e.g., George et al. 2020, Dunn, 2018; Corley, 2021) *or* by climate conditions (e.g., Amegbor et al., 2020; Baker & Anttila-Hughes, 2020; Niles & Brown, 2017; Thiede & Strube, 2020) and rarely directly consider situations where conflict events and climate extremes occur together (Delbiso et al., 2017 and Rowhani et al., 2012 provide notable exceptions).

These distinct areas of research use a range of different methods and datasets to explore the idea that climate or conflict factors disrupt the food system and may lead to an increase in household-level food insecurity and malnutrition. However, it is not clear what happens to food security and child health when a poor agricultural season occurs at the same time and in about the same place as a violent conflict event (Justino, 2011). In other words, when violent conflict occurs during a poor growing season does that strain the food system and increase food insecurity even further than if either a drought or a conflict occurred alone? Furthermore, can we gain insight into the spatial and temporal variation in acute childhood malnutrition—an outcome of food insecurity and an important underlying component of mortality—when we consider the relationship between WHZ, armed conflict, and local climate factors together?

In this analysis, we evaluate the relationship between individual-level WHZ and recent community-level climate conditions, growing season conditions, and regional-level conflict conditions. In an effort to advance understanding of how these relationships may vary depending on the setting, we compare results across three sub-Saharan African countries with unique histories of conflict: Nigeria, Kenya, and Uganda. The countries share important characteristics relevant to this analysis, including relatively high and persistent rates of acute malnutrition, as well as spatially varying land use and climate and conflict conditions. We conduct a comparative analysis across and between countries to allow for discussion of both the general trends that might explain variation in WHZ while exploring context-specific relationships between conflict, climate and child health.

To conduct this analysis, we use several rounds of individual-level, spatially referenced cross-sectional survey data from recently collected Demographic and Health Surveys (DHS), providing large and nationally representative samples of WHZ. Violent conflict data come from the Uppsala Conflict Data Program (UCDP) and capture sub-national, regional-level lethal events in the context of violent conflict involving organized armed actors (state and/or non-state forces). Climate data come from several different sources capable of capturing relatively fine-scale variation in conditions over time and space. Regression analyses that consider a range of individual- and household-level factors in addition to the climate and conflict conditions are estimated for each country.

## 2 Background

### 2.1 Acute Malnutrition

Acute malnutrition occurs because of sudden and rapid change in nutrition and caloric intake and can be identified through wasting or a reduction in a child’s WHZ. According to recent global estimates, 17 million children under age 5 were categorized with severe acute malnutrition and another 32 million were categorized with moderate acute malnutrition (UNICEF/WHO/World Bank, 2019).^1^ Differing from other forms of malnutrition (e.g., stunting), treatments exist that can move children out of the wasted categorization and into a healthy weight for their height (UNICEF/WHO/World Bank, 2019). Because wasting increases mortality risk, but can also be successfully treated, researchers and policymakers continue to work to identify different risk factors associated with wasting to support effective interventions. Through empirical research and policy efforts, a number of broadly relevant and widely used frameworks have emerged to help identify key areas for interventions (Hall et al., 2011; Kandala et al., 2011; Bhutta et al., 2017, UNICEF/WHO/World Bank, 2019). In particular, the popular framework of the United Nations Children’s Fund (UNICEF) organizes factors into three categories: (1) *immediate causes* involving dietary intake and disease at an individual level, (2) *underlying causes* related to food security, care practices, and hygiene environments at household or community levels, and (3) *basic causes* such as political and economic conditions, land use, and education, at sub-national regional, country or international levels (UNICEF/WHO/World Bank, 2019).

While the UNICEF framework reflects an integration of different factors across scales, existing research has typically focused either on more micro-level factors (as in categories 1 and 2) *or* on macro-level factors (as in category 3). Projects that focus primarily on the macro-level patterns risk over-generalizing and may miss important within-group heterogeneity (Arora-Jonsson, 2011; Hallegatte & Rozenberg, 2017), while those, often smaller-scale, studies that focus primarily on individual-level variation may struggle to identify the underlying and basic factors associated with child health and to situate their findings beyond the specific research setting (Prado et al., 2019, Phalkey et al., 2015). A rapidly emerging body of literature aims to consider the individual within context, with attention to how contextual factors measured at different spatial scales may be useful for understanding the complex and multi-scalar processes underlying individual-, community- and regional-level health variation (e.g., Amegbor et al., 2020; Corley, 2021; Delbiso et al., 2017; Rowhani et al., 2012; Thiede & Strube, 2020; Thiede et al., 2020). This paper seeks to contribute to this emerging literature.

### 2.2 Conflict Events, Climate Conditions and Child Health

Conflict and climate conditions are regularly linked to food security, nutrition, and health outcomes, including wasting, using the UNICEF and other related frameworks (Thiede & Strube, 2020; Phalkey et al., 2015; Brown et al., 2020; Corley, 2021; Justino, 2011; Hill et al., 2019). The specific pathways and linkages explored when evaluating these different external factors and outcomes vary because of different data, analytic goals, and disciplinary perspectives. In general, researchers propose that adverse climate conditions reduce cropped and harvested area and also reduce agricultural yields. Conflict conditions may create significant challenges when individuals and families try to buy or sell food and livestock from local markets (Justino, 2011, Verpooten, 2009, Corley, 2021). In terms of specific impacts on child health, researchers find that warmer, dryer climate conditions in sub-Saharan Africa are generally associated with adverse child health outcomes in terms of wasting, stunting, or related nutrition outcomes (Cooper et al., 2019, Thiede & Strube, 2020, Davenport 2017). Empirical studies have detected greater rates of chronic malnutrition among children in settings affected by armed conflict in several African countries, including Burundi (Bundervoet et al., 2009), Ethiopia and Eritrea (Akresh et al., 2012), Cote d’Ivoire (Minoiu & Shemyakina, 2014), and Rwanda (Akresh et al., 2011) and increased risk of acute malnutrition in Nigeria (Dunn, 2018; Howell et al., 2018) and Somalia (Kinyoki et al., 2017). Research of armed conflict and agricultural labor demands shows that separately these factors may adversely impact the care environment with a negative impact on food and nutrition outcomes (Betancourt & Khan, 2008; Gates et al., 2012; Mansour & Rees, 2012; Randell et al., 2020, 2021).

Depending on the type of data used and the specific research design, considering place-based factors related to conflict and climate helps to situate individuals within their unique contexts, as in the UNICEF structure, while also allowing for discussion of relationships between variables (Amegbor et al., 2020; Corley, 2021; Prado et al., 2019). Integration across scales implies that not all households or individuals exposed to the same contexts or macro-level conditions will necessarily experience the same health outcomes. This approach also suggests that how factors are defined and measured in each level of the UNICEF framework may also be dependent on the place and time (Harris and Nisbet, 2020, Prado et al., 2019; Amegbor et al., 2020; Rowhani et al., 2012). What this means in practice is that the way conflict and climate factors relate to individual-level WHZ and wasting outcomes may vary over space and time and may be exacerbated or mitigated by dynamic conditions (e.g., disease or available resources).

One of the ongoing discussions in climate-health and conflict-health research is using appropriate spatial and temporal aggregation strategies. In fact, related research uses vastly different aggregation approaches when considering these exposures (see Phalkey et al., 2015, Buhaug, 2015; Jia et al., 2019; Thiede et al., 2020; Rowhani et al., 2012; Delbiso et al., 2017). Therefore, ambiguity still exists about the ideal time frame for considering climate or conflict impacts on child health. Recent efforts in a small selection of countries have highlighted that for food security outcomes, locally occurring slow-onset events like drought will generally begin to modify household consumption and impact some nutrition outcomes within about 5–11 months after the growing season (Hill et al., 2019). For rapid-onset disasters (like earthquakes), the timing and spatial footprint of the impact will vary based on magnitude of the event and the impact on local infrastructure and the presence of factors like humanitarian aid. The impact from violent conflict on individual-level nutrition may occur without much of a temporal lag but will depend on local and individual-level vulnerabilities (Hill et al., 2019).^2^ In all cases, timing, spatial extent, and local vulnerabilities are important to consider to advance the scientific understanding of the processes underlying acute malnutrition and to help identify the key windows for humanitarian interventions to reduce morbidity and mortality among those with the greatest needs.

Here we consider climate factors at the scale of the community (10 km)—a finer-scale of analysis that acknowledges that spatial heterogeneity in rainfall, temperature and growing season conditions in the three countries. Much of the related research uses significantly coarser data, approximately 5–10 times coarser than our climate measures (e.g., Thiede & Strube, 2020). We also employ a relatively extreme measure of conflict: violent armed conflict associated with a lethal event at the regional-level scale (subnational administrative level 1). Other approaches used in the literature vary dramatically and include considering all reported events, regardless of magnitude, using binary presence-absence indicators (e.g., Delbiso et al., 2017; Dunn, 2018) versus those approaches that focus only on significant levels of battle-related deaths (> 25 per annually) (Rowhani et al., 2012). We choose a measure of conflict that falls somewhere between these two extremes—at least one fatality from a violent event—and count the number of these events within the region. The reason for this measure is because armed conflict leading to a lethal event may be more likely to change behaviors and access to agricultural plots and markets, and result in reduced food availability or access. Using an event count, rather than a binary (yes/no) indicator may also allow us to consider the intensity of exposure to conflict in an area as well.

In terms of the temporal dimension, most variables are collected at the time of survey for each individual child (between ages 24–59 months of age). Given the potential for temporal lags between conflict events or the growing season and malnutrition outcomes, we match children to local conditions with attention to specific time frames. WHZ is sensitive to very recent changes and children are able to regain weight lost, we focus on conflict conditions that occur relatively close in time to the survey date (within 6 months). We further explore the conflict-WHZ relationship using two different time lags—1–3 month and 4–6 month lags—of the count of events of violent conflict within the region. The idea is that impacts on acute malnutrition of violent conflict could appear very soon after the event or after a short delay if food and health services are interrupted for several months. We also consider the quality of the most recent growing season as part of the potential for a sub-optimal growing season to increase food insecurity. Finally, because climate conditions like flooding and high temperatures can have immediate impacts on WHZ (Delbiso et al., 2017; Thiede & Strube, 2020), we include average climate conditions of the 3 months before the survey. Our approach aims to advance discussion of the individual-level vulnerabilities, specifically with how recent conditions relate to acute malnutrition, in a context of climate change and in the presence of violent conflict. Including factors at different scales and time periods provides insight into the questions of which sub-national geographic areas—and within those areas, which specific households and children—are more likely to experience acute malnutrition.

## 3 Setting

In this study, we include three African countries: Kenya, Nigeria, and Uganda. In all three countries, child malnutrition, food security, and land use vary spatially (Akombi et al., 2019; Amegbor et al., 2020; Grace et al., 2012). Each country has a history of armed conflict, exhibits a food system significantly dependent on locally grown, rain-fed crops, and faces persistent child malnutrition. In Nigeria, the southern regions are hot and wet most of the year with a long wet-season, whereas regions in the north having an extended dry season from October to April. In Kenya, the long and short rains characterize the highly populated temperate subtropical climate in the southwest highlands, and a much dryer climate in the north and east. Similar to Kenya, Uganda has two rainy seasons, but is in general semi-humid with some regions receiving up to 2200 mm of rain (see FEWS NET livelihood zone descriptions for more details for within-country variation).

Conflict also varies spatially and temporally within each country—with the armed conflict events observed in this study outcomes of different political events, state-sponsored violence and within-country dynamics between groups (see Thiesen et al., 2012, Adano et al., 2012) Kenya has been comparatively peaceful with some recurrent conflict events in the North and Northeast (Thiesen et al., 2012). In Uganda—especially the North and Eastern areas of the country—significant violent conflict has been present since the 1960s up through at least 2010 (Kandel, 2016). In Nigeria, the year 2000 brought a shift in political leadership and increasing tension between religious groups resulting in increasing violent conflict events across much of the country with Boko Haram becoming more violent towards civilians beginning about 2010 (Dorff et al., 2020; Dunn, 2018).

## 4 Methods and Measures

### 4.1 Data

In the analysis, we use a combination of cross-sectional and time-varying data drawn from multiple sources, summarized in Table 1. The DHS provides cross-sectional data on anthropometric measures for children and other indicators of health, demographic, and socio-economic characteristics of individuals, families, households, and communities. We use multiple waves of the DHS that were conducted in Kenya (2003, 2008, 2014), Nigeria (2003, 2008, 2013) and Uganda (2006, 2011, 2016). We include all children surveyed between the ages of 24–59 months. The reason we use this age range is because the DHS contains the most detailed information on child health for children under 60 months old. We exclude the youngest children (under 24 months) even though wasting levels may be relatively high within this age group. We exclude this age group because breastfeeding behaviors may vary significantly with notable impacts on child health (Randell et al., 2021). The DHS information on breastfeeding does not contain sufficient detail to evaluate frequency, duration, and other care factors vital to growth of children under 24 months.

The Uppsala Conflict Data Program’s Georeferenced Event Dataset (UCDP-GED) Version 19.1 (Högbladh, 2019; Sundberg & Melander, 2013) supplies the data on conflict. UCDP-GED comprehensively captures events of organized armed violence that occurred during conflicts active around the world since 1989. An event is “an incident where armed force was used by an organized actor against another organized actor, or against civilians, resulting in at least one direct death at a specific location and a specific date” (Högbladh, 2019). The dataset includes the georeferenced location and specific date of each event, the type of the conflict (state-based conflict, non-state conflict, or one-sided violence), the actors involved, the severity (measured in terms of battle-related fatalities), and other descriptive information. In the related literature on health and conflict previously cited, a range of different conflict measures are employed and explored for different purposes and with no clear consensus on measures (see Eklund et al., 2017 and Koren et al., 2018). We specifically focus on armed conflict and lethal events as a relatively narrow and severe form of conflict (versus cattle raids or urban demonstrations, for example) with the idea that this type of conflict may be the most disruptive to local agriculture and food systems. Spatial details on the location of conflict events are varied, in part because of the data collection process. Ultimately, the finest reliable spatial detail for the data is the region-level (subnational administrative level 1). Please see a longer description in the “Appendix” about the spatial detail of the conflict data.

The Climate Hazards InfraRed Precipitation with Stations (CHIRPS) dataset provides daily precipitation at a 0.05° × 0.05° degree spatial resolution (~ 5 km^2^) around the world (Funk et al., 2015). US and international agencies (e.g., Famine Early Warning System Network (FEWS NET)) routinely use the CHIRPS data set to monitor drought and food insecurity. Also, the recently developed Climate Hazards InfraRed Temperature with Stations (CHIRTSmax) dataset provides daily maximum temperature with the same spatial resolution and global coverage (Funk et al., 2019, Verdin et al., 2020). This resource, which leverages remotely sensed infrared radiation temperatures to estimate two-meter air temperatures, is demonstrated to have excellent measurement performance, most notably in data-sparse regions such as sub-Saharan Africa (e.g., see usage notes in Funk et al., 2019; Tuholske et al., 2021). We rely on CHIRPS and CHIRTSmax to derive spatially and temporally varying indicators capturing climate conditions near to the date of interview.

We also use annual seasonal maximum of the Normalized Difference Vegetation Index (NDVI) from NASA’s Moderate Resolution Imaging Spectroradiometer (MODIS). This fine-grained measure of vegetation vigor allows for a direct measure of above ground biomass as related to agro-climatic growing conditions during the primary growing season (as indicated by FEWS NET’s growing season calendars). NDVI describes the state of vegetation and has been shown to correlate well with deviations in crop yield, though performance is variable by region (Vrieling et al., 2008). A common practice is to use community-level aggregate NDVI as a proxy for local-scale crop health and agricultural production of food (Petersen, 2018, Teal et al., 2006, Brown & de Beurs, 2008, Shukla et al., 2021, Grace et al., 2021).

We use livelihood zone data from the Famine Early Warning System (FEWS NET). These zones capture general food and income characteristics of an area (http://fews.net/). The FEWS NET zones provide more detail (on specific types of crops and livestock, for example) than is necessary for this project and we instead, aggregate the zones into broad categories. The categories themselves and zone aggregations vary somewhat by country, in our case the Uganda zone includes more information on “urban” areas than the other two countries, for example, but in general they differentiate between those households with a greater dependence on cropping (agriculturalists), from those that are more likely dependent on livestock (pastoralists), from those engaged with both livestock and crops (agropastoralists). The FEWS NET zone reports also provide information on the key growing season months for each country used to calculate seasonal maximum NDVI.

Aggregated livelihood zones and growing season calendars have been used in studies of children’s health and climate in the developing world (e.g., Bakhtsiyarava et al., 2018; Davenport et al., 2020; Hill et al., 2019; Randell et al., 2021). While not everyone within a particular zone is equally engaged in the same strategy to procure food or income, the data provide a way of gauging differences among geographic areas that may condition the impact and timing of climatological and conflict factors on economic and food production strategies.

### 4.2 Spatially Merging Data

The DHS is spatially referenced at the level of the survey cluster. Global Positioning System (GPS) coordinates are collected and publicly released for locations of clusters where surveys are administered. Each cluster consists of a number of households that participated in the survey, spread across one or more populated places within a geographic area. To maintain confidentiality, the location of the survey cluster is randomly displaced. Most coordinates for clusters in rural locations are displaced by up to 5 km in any direction; a random sample of 1% of rural clusters are displaced by up to 10 km. For urban clusters, the displacement is up to 2 km.

**Fig. 1:**
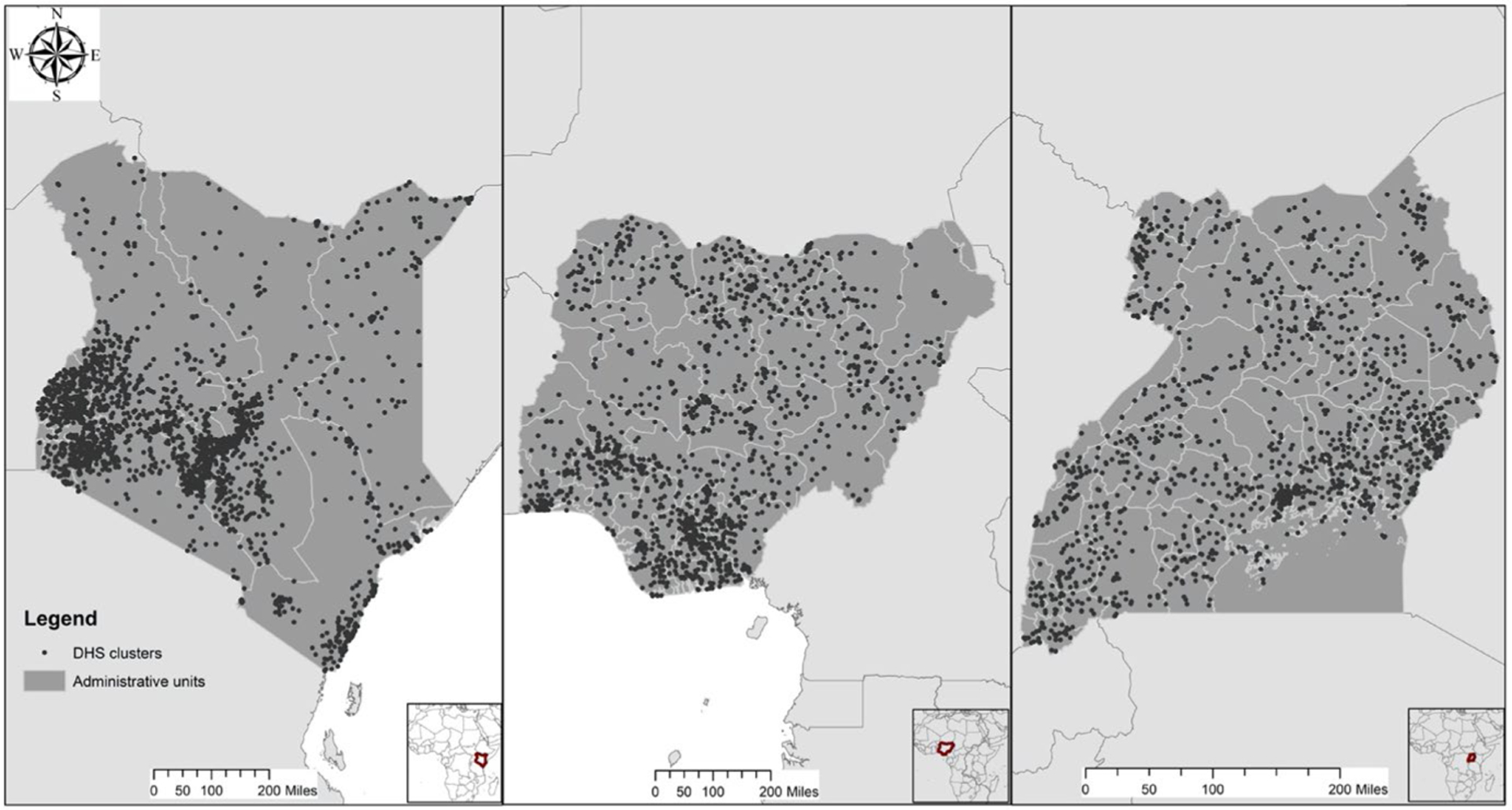
DHS clusters and regional boundaries (first-order administrative division) for Kenya, Nigeria, and Uganda (in order)

DHS recommends that researchers average any environmental data over a 5–10 km buffer around the coordinates of each DHS rural cluster, with the expectation that the specific community where households were sampled will fall somewhere within this buffer (Perez-Heydrich et al., 2016). The three environmental indicators – rainfall, temperature, and NDVI—are calculated by averaging across the area (using a 10 km buffer) around each offset DHS survey cluster GPS point. As previously, noted, considering climate/environmental variability at the level of the DHS community allows for comparisons across communities (Randell et al., 2020) and differs from some of the more coarse-scale analysis, providing greater insight into the “on-the-ground” conditions (Tuholske et al., 2021). The cluster’s offset GPS point is also matched to regional and livelihood-level boundaries to merge with the data on conflict and livelihood zones. For merging with administrative regions or livelihood zones, we use whichever region or zone contains the publicly released point. In Fig. 1, we show maps of the DHS clusters and include regional boundaries for each country.

### 4.3 Measures

Table 2 summarizes the data, by country, for all the variables used in our analysis. Variables are grouped by the level of measurement in the multi-scalar structure of the analysis. For each variable, we report means or percentages as appropriate. The dependent variable in our analysis is a child’s WHZ from the DHS data.

We also include a range of independent variables that map onto the levels of the UNICEF framework. Our choice of variables is based on findings from related research that have identified specific factors as associated with acute malnutrition, and are organized using the UNICEF framework’s categories of basic, underlying, or immediate causes (e.g., Hill et al., 2019; Randell et al., 2020,)

#### 4.3.1 Key Variables in the Analysis (Associated With Basic Causes)

We include multiple indicators reflecting the *count of conflict events*, all of which are derived using the UCDP-GED data. To address variability in the spatial precision of the measurement of event locations, we aggregate counts at the level of the region. Aggregations are performed with reference to the most recent regional boundary data obtained from the Integrated Public Use Microdata Series—International (IPUMS-I) for the relevant boundaries used in the UCDP-GED for Kenya 2009 (provinces), Nigeria 2010 (states), and Uganda 2002 (districts). We consider counts of armed conflict events during the 1–3 month and 4–6 month intervals before the month of the DHS data collection for each child. Empirical analyses of conflict impacts use widely varying conflict measures (see Rowhani et al., 2012; Delbiso et al., 2017; Buhaug, 2015). While there is no consistent time period of conflict exposure and child health in the literature, some researchers consider cumulative effects, impacts within the last 6–12 months or the most recent month to the survey. Building on the discussion in Hill et al. (2019) comparing rapid onset and slow onset events, we choose these two different (1–3 month and 4–6 month), yet relatively proximate, time frames to help uncover the temporal process that connects conflict to child health shortly after exposure to conflicts (Hill et al., 2019).

To measure climatological variation that may have a direct association with child health, we include two variables that measure the *average total precipitation* and the *average maximum temperature* (derived from CHIRPS and CHIRTSmax) during the three months prior to the data collection in a given survey cluster (see Randell et al., 2020; Rowhani et al., 2012; Grace et al., 2015).

As a gauge of agricultural productivity, we include the *maximum NDVI* for the most recent completed primary growing season in each country (e.g., Bakhtsiyarava et al., 2018). Growing season information is obtained from the FEWS NET country-specific growing season calendars. NDVI values range from −1 (no vegetation/greenness) to 1 (high indication of greenness/vegetation). In general, more vegetation (higher NDVI values) are associated with more a better growing season and more agricultural productivity (Shukla et al., 2021). In addition, we include dummy variables that flag whether or not any conflict during the periods measured by the lagged variables coincided with the growing season.

#### 4.3.2 Other Independent Variables Associated with Underlying Causes

We include several variables to control for potential underlying causes vulnerability to acute malnutrition. The *child’s age*, *sex,* and *birth order* (first-, second-, third-born, etc.) are routinely included in studies of child health and food security because they capture biologically and culturally relevant factors related to growth, feeding, and caretaking. We also include child’s birth month and birth year. Consistent with related research we also include *maternal age* and *educational attainment* (Corley, 2021; Prado et al., 2019; Thiede & Strube, 2020). Another set of variables measures characteristics of households. Household *toilet facilities* and *water sources* are associated with malnutrition in some settings possibly through pathogens in fecal matter and contaminated water (van Cooten et al., 2019). The *flooring material* in the house where a child currently lives (either unfinished or finished) provides a simple indicator to approximate a household’s socio-economic status, primarily by differentiating the most poor (those with unfinished flooring) from the less poor (those with finished flooring). Household electricity-status may capture food storage potential as well as general socio-economic status and is also included in the analysis. Flooring has the advantage of tending to be less correlated with urban or rural residence than other factors that can capture aspects of socio-economic status such as electricity use or water access (Davenport et al., 2017; Dunn, 2018). The household-level variables are relatively static, compared to other measures of socio-economic status that focus on different types of assets or especially on income. Thus, the value of each of these variables when the survey was administered is likely to reflect conditions in at least the recent past, potentially dating back to when the children in the household were born.

We also include characteristics of communities (i.e., survey clusters). Whether the location is *urban or rural* (a classification determined with reference to criteria specific to each country) and the *type of livelihood zone* (agricultural, agropastoral, fishing, pastoral, or urban, from FEWS NET) defines a surrounding context that can influence food security and health outcomes. These indicators capture broader temporal trends and spatial patterns that can likewise affect food security and health outcomes, beyond what is reflected in other variables. We include region of residence to control for regional-level time invariant factors.

#### 4.3.3 Other Independent Variables Associated with Immediate Causes

Finally, we consider whether or not each child experienced fever and/or diarrhea within the 2 weeks preceding the administration of the survey, using the data collection in the DHS. Both of these variables are known to be proximate risk factors for acute malnutrition (Tesfai et al., 2013; WHO, 2017).

### 4.4 Statistical Analysis

We estimate a suite of regression models to explore the relationships between conflict, climate and acute malnutrition. The primary model is a linear mixed-effects regression model estimating the continuous WHZ score for each child in relation to the number of conflict events in the child’s region of residence. The model also includes immediate and underlying factors that can impact child WHZ as described above.

The data has a hierarchical structure^3^—there are often several children with the same mother and there are several households within the same DHS cluster. To account for the shared characteristics between individuals, we include a random effect for the mother and for the DHS cluster (Currie & Schwandt, 2013; Gelman & Hill, 2007).

The model can be specified as follows:

$$\left(Y_{ijk}\right)=\beta_{0}+\beta_{1}\left(conflict_{jk}\right)+\beta_{2}\left(conflict\_grs_{jk}\right)+\beta_{3}\left(temp_{jk}\right)+\beta_{4}\left(precip_{jk}\right)+\beta_{5}\left(NDVI_{jk}\right)+\beta_{n}\left(X_{z}\right)+w_{j}+u_{k}+e_{ijk}$$

In the equation, Y is a continuous WHZ score for child *i* from mother *j* in DHS cluster *k*. *Conflict* is a count variable counting violent events (within an administrative region) during either a) 1–3 months before the survey or b) 4–6 months before the survey. We estimate separate models for the number of conflict events during the 1–3-month period before the survey (Model 1) and 4–6-month period before the survey (Model 2).^4^ *Conflict_grs* is a dummy variable—a value of 1 indicates that when conflict occurred it occurred during the growing season and a value of 0 indicates the conflict did not occur during the growing season.^5^ Parameters *β*_3_, *β*_4_, and *β*_5_ are the terms for the maximum average monthly temperature three months before the survey, average monthly precipitation three months before the survey, and maximum NDVI during the previous growing season, respectively. Keep in mind that these variables are dependent on the interview date (of the mother) and the cluster location. The model also controls for child-, mother-, household-, and community-level variables (*X*_*z*_) described in the Measures section, as well as fixed effects for the month and year of birth and region of residence. *W*_*j*_ is a random effect for woman, and accounts for multiple children per woman. The DHS cluster-level random effect is specified by *u_k_*. Fixed effects account for unobserved (time invariant) factors.

## 5 Results

We first describe the overall results by country below. We then summarize the patterns that we observe in terms of timing of conflict and spatial variation when considering the results together.

### 5.1 Kenya

The frequency of armed conflict events within the administrative region where a child lives during the 1–3 month lagged period is not significantly associated with a child’s WHZ score as shown in Model 1 in Table 3. However, in the case that the timing of the specific conflict occurred during the growing season, we observe a significant negative relationship between the exposure and WHZ score. Thus, when considering conflict events during the 1–3 month period, the count of events itself was not significant, however if conflict occurred during the growing season, the mean WHZ score is 0.06 standard deviations lower than if the conflict did not occur during the growing season. In the event that conflict occurred 4–6 months before the survey month, Model 2, we observe a significant and negative association with child health but no linear association with regard to growing season timing of this later lag. An additional conflict event is then associated with a 0.01 decrease in mean WHZ score. In terms of climate and environmental conditions, poor growing conditions, as indicated by lower amounts of precipitation, higher maximum temperatures, and a lower vegetation (NDVI) index, are negatively associated with WHZ scores. These relationships remain consistent across the two models.

Household- and mother-level patterns suggest that, in general, children living in households with more resources (e.g., finished flooring, electricity, higher maternal education) have, on average, higher WHZ scores than children in less well-resourced households. Child-specific factors also play an important role, with younger children who are of a higher birth order (in other words, children with more siblings), tending to have lower WHZ scores. Factors associated with recent illness—diarrhea and fever—are also negatively associated with WHZ.

### 5.2 Nigeria

According to the Models for Nigeria (Table 4), children who live in regions of Nigeria that experienced higher counts armed conflict within 1–3 months or 4–6 months before the survey are more likely to have lower WHZ scores. In Model 1, conflict occurring during the growing season is associated with lower WHZ scores—a reduction of 0.84. The results also show that greater amounts of precipitation and higher temperatures are associated with reduced WHZ scores. While elevated temperatures are commonly associated with worse health outcomes (Thiede & Strube, 2020), rainfall is often positively associated with health outcomes, although not consistently (Grace et al., 2021; Randell et al., 2020; Thiede & Strube, 2020). A possible explanation for these relationships is that hot and wet conditions may be associated with disease—especially malaria. Therefore, it is possible that the recent weather conditions are indicative of increased risk of malaria among children. In Kenya, by contrast, malaria is not consistently endemic across the country and precipitation conditions are more likely associated with high quality growing seasons and higher food availability.

In terms of household-, maternal- and child-level factors, a child with a recent fever in Nigeria is significantly more likely to have a worse WHZ score than a child who has not had a recent fever. We also observe a general increase in WHZ for female versus male children. Other factors associated with household or community resources are not significantly associated with the outcome variable—possibly suggesting that these community- and regional-level factors are important for understanding variation in risk of acute malnutrition in Nigeria.

### 5.3 Uganda

As reported in Table 5, no statistically significant association was detected between the frequency of recent conflict events and WHZ scores among children in Uganda, even when conflict occurred during the growing season. As mentioned in the Methods section—we also estimated models where we combined the counts of conflict over the prior 6 months and where we used an NDVI × conflict interaction effect (see “Appendix“). We do not present all of these results—but they all generally produced estimates yielding similar insight—there is no significant relationship between conflict, environmental factors and WHZ. None of the environmental factors were significantly associated with a child’s WHZ score. Beyond living in a pastoral region, where children, on average, have WHZ scores that are around 0.40 standard deviations lower than children living in agricultural areas, the only factors with significant associations are select immediate and underlying causes. Specifically, children who are younger, female, experienced a recent fever, live in households with piped water (versus relying on surface water or sources that may include water trucks and purchased water) and no electricity are more likely to have lower WHZ scores than their counterparts.

### 5.4 Similarities and Differences Across the Three Countries

Across the three countries and during similar time frames, we observe very different relationships factors associated with variation in WHZ. The relationship between counts of conflict in the administrative region of residence, and WHZ varies significantly across the settings. We note that there is a significant negative relationship between the count of events and WHZ score in Nigeria versus non-significant correlations in the other two countries. Variability, however, is relatively higher in Uganda than in Kenya. The environmental factors of interest also vary by country with Kenya and Nigeria reporting significant impacts on WHZ from rainfall and temperature (albeit the coefficients are very small and the direction of the relationship varies for rainfall). NDVI is positively and significantly related to WHZ scores in Kenya and not significant in the other two countries. In general, despite their geographic proximity, results from Uganda and Kenya, appear quite different from each other in terms of the significance of household-, maternal-, and child-level factors with very little obvious shared relationships between the conflict and environmental factors. Considering the regression results together, therefore, suggests that exposure to conflict is related to child health but only as it acts through other downstream factors and suggests that there are a number of unmeasured factors that differentiate the contexts. In other words, there are additional place-based factors that must be considered that shape the way that conflict relates to health outcomes.

Among the other factors included in the analysis, there are very few similarities across the countries in terms of significance, or magnitude and direction of factors. For example, a child’s age and sex are significant across all three models (although in Kenya, sex is not significant). However, in Nigeria older female children have higher WHZ scores whereas in Uganda, younger male children have higher WHZ scores. In Kenya, younger children, regardless of sex, have lower WHZ scores.

## 6 Discussion

Unlike other forms of malnutrition, with strategic interventions, acutely malnourished children can recover and avoid serious illness and death. To combat acute malnutrition and lessen the adverse impacts of this food insecurity outcome, researchers and policymakers continue to work to uncover the processes associated with acute malnutrition and variation in WHZ. It is therefore especially important to consider the timing of exposure and how different contexts shape the relationship between exposure and outcomes.

Broadly speaking, our results suggest varied relationships between conflict, climate and food security across the three countries. In other words, the linkages between climate and conflict and acute child health outcomes related to food insecurity and malnutrition, are not consistent across settings. At the country-level, however, there are clear indications for Kenya and Nigeria, that targeting interventions based on climate and conflict conditions could help to improve child health and reduce wasting. Developing effective interventions will likely require ongoing monitoring and evaluation at a sub-national-level, using detailed spatially relevant data that can capture a range of local factors.

Our analyses employed fairly fine-scale measures of climate conditions associated with child health—rainfall and temperature conditions around the time of survey—as well as with vegetation measures associated with local agricultural production (seasonal NDVI—a measure of growing season vegetation). As compared to related research (e.g., Thiede & Strube, 2020; Baker & Antilla-Hughes 2020; Dunn, 2018), these climate and vegetation measures employ more spatial variation. Despite these highly detailed data, however, the relationship between the variables and the outcome are not consistent across settings. We interpret this finding as suggesting that there are local strategies that individuals, households or communities employ to secure their children’s health in the context of specific climate and food production conditions. These strategies are potentially situated in historic and place-based cultural practices associated with risk management that are not measured in the standard health surveys used here yet are key components of vulnerability and variability in child health outcomes in a context of climate change. Locally varying factors, unmeasured in this analysis, related to roads, humanitarian aid, population mobility, among other factors may also play an important role. Future research exploring these dimensions may help to isolate these different relationships (Corley, 2021; Dunn, 2018).

Along the same lines, the relationship between armed conflict and acute malnutrition is inconsistent across countries and time periods. While this finding mirrors related research that fails to find a consistent linkage between conflict and food security, our approach suggests that even when survey data from the same source as well as constructing identical measures of conflict across settings, the relationship between conflict and acute malnutrition is not consistent across countries. While some research has suggested that there may be some variation in the relevant timing of the events on child health outcomes and food security, the results here suggest that there is not a consistent temporal linkage between events and child health outcomes. We attempted to model conflict using a relatively extreme definition of conflict, one which may be most likely to impact food systems and change behaviors (Ghobarah et al., 2004), nonetheless, in the case of Uganda we see no relationship between conflict conditions and child health. Whereas in Nigeria we see a significant association between conflict and child health, with WHZ scores decreasing greater when conflict events occurred during the growing season. In Kenya, however, the relationship is somewhat different, with a significant association between conflict and child health in the case that a child is exposed to conflict during the 3 months preceding the survey and if this period occurs during the growing season.

We are unable to evaluate how the historical presence of conflict has shaped the ways that communities cope with armed events. Kenya has been comparatively peaceful compared to the other countries, Uganda, has become more peaceful after an extended civil war, while Nigeria has just entered into a situation of escalating violence. Is it possible that Ugandans are accustomed to violence in a way that reduces the potential for violent events to dramatically uproot behaviors and experiences? The data available and our analytic approach was unable to uncover the ways that historical experiences shape current realities.

Additionally, the analysis could examine conflict in a more spatially specific manner (using finer-scale data on conflict locations and durations), similar to what is possible with climate. Doing so might help reinforce our ability to determine if individual children and households in specific neighborhoods have been exposed to observed conflict conditions. A limitation of the available data, however, is that precision in the measurement of spatial locations of conflict events is reliable only to the regional administrative level. Some conflict is actually dispersed to that extent, affecting larger areas within a country. Other conflict events are simply measured imprecisely, given available information. Whether conflict is dispersed within a region, or merely occurred within a region but was not necessarily nearby a specific household, a potential exists for residents of the region to be exposed to the implications.

We also note that there are important limitations with the health survey data included in this research. One limitation is that we focus solely on living children and living respondents (mothers)—perhaps our sample includes those who are the most robust as the frailest have not survived to be included in the survey. This kind of bias would lead to results showing that the effects of conflict are less severe than they actually are. Additional research using spatially detailed data on maternal and infant/child mortality would help to uncover the potential for selection bias. Overall, our research highlights the need for improved data on armed conflict that can be used within the context of individual-level studies on nutrition and food security. It also highlights the need for more qualitative investigations that consider household coping strategies reflecting the multiple stresses that families may face in a context of climate change.

## Figures and Tables

**Table 1:** Overview of data sources

Dataset	Uses	Spatial resolution of the raw data	Temporal resolution in analysis
The Demographic and Health Survey (DHS): Kenya (2003, 2008, 2014); Nigeria (2003, 2008, 2013); Uganda (2006, 2011, 2016)	Individual-level health and socio-demographic characteristics	2–10 km	Month of data collection for each observation (individual child aged 24–59 months)
Uppsala Conflict Data Program Georeferenced Event Dataset Version 19.1	Count of lethal events resulting from armed conflict	Region (First-order administrative division)	Separate three-month intervals (1–3 vs. 4–6) prior to month of data collection for each DHS observation (individual child)
Climate Hazards Group InfraRed Precipitation with Station (CHIRPS) data	Precipitation	0.05 degree	Total monthly precipitation averaged over three months prior to month of data collection for each DHS observation (individual child)
Climate Hazards Group InfraRed Temperature with Station (CHIRTSdaily) data	Temperature	0.05 degree	Maximum monthly temperature averaged over three months prior to month of data collection for each DHS observation (individual child)
MODIS Normalized Difference Vegetation Index (NDVI)	Vegetation greenness measure capturing growing season quality or livestock grazing potential	250 m	Maximum value from the most recent growing season (defined by FEWS NET calendars)
FEWS NET zones And growing season calendars	General food and income acquisition strategies of an area and timing of key growing seasons	varies	Varies by country (developed at a single point in time for each country)

**Table 2:** Summary information of variables included in the analysis

Variables	Kenya		Nigeria		Uganda	
	Men	%	Men	%	Men	%
*Weight-for-height z-score*	− 0.18		− 0.27		0.13	
*Sub-national regional (ADMl) level*						
Conflict events 1–3 months prior (count)	3.32		1.07		0.2	
Conflict period overlaps with growing season (yes)		35		47		89
Conflict events 4–6 months prior (count)	2.59		1.09		0.19	
Conflict period overlaps with growing season (yes)		30		49		82
*DHS Survey cluster (community) level*						
Temperature degrees Celcius (average maximum temperature 3 months before survey)	29.35		35.36		28.89	
Precipitation (mm) (average maximum temperature 3 months before survey)	93.89		59.93		118.03	
NDVI (during previous growing season)	0.65		0.57		0.72	
Rural place of residence		72		67		84
Livelihood zone (agricultural)		50		70		25
Agroplastoral		28		16		58
Fishing		7		11		5
Pastoral		15		3		2
Urban*		–		–		10
*Household level*						
Toilet facility (none)		23		31		12
Flush		8		14		2
Non-flush		69		55		86
Drinking water (piped)		29		10		16
Well		25		61		65
Surface		42		24		18
Other		4		5		1
Finished floor		32		60		24
Electricity		18		46		17
*Mother level*						
Age (years)	29.63		30.39		30.03	
Education (none)		21		46		17
Primary		55		23		63
Secondary +		24		31		20
*Child level*						
Age (months)	40.97		40.92		40.85	
Female		50		50		50
Birth order	3.5		3.95		4015	
Recent diarea (yes)		11		9		16
Recent fever yes)		25		15		40

* For Uganda, We use the livelihood zone coding for urban versus rural.

For Kenya and Nigeria we use the DHS coding for urban

**Table 3:** Climate and conflict continuous WHZ score regression results for children aged 24–59 months in Kenya

Independent variables	Model 1			Model 2		
	Coefficient	Confidence Interval	p-value	Coefficient	Confidence Interval	p-value
*ADM1 Level*						
Conflict events 1–3 months prior (count)	−0.01	(−0.01, 0.00)	0.21			
Conflict period overlaps with growing season	−**0.06**	(−0.12, −0.00)	**0.04**			
Conflict events 4–6 months prior (count)				−**0.01**	(−0.02, −0.001)	**0.03**
Conflict period overlaps with growing season				−0.04	(−0.10, 0.02)	0.22
*Community Level*						
Temperature (average maximum temperature 3 months before survey)	−**0.02**	(−0.03, −0.01)	<**0.01**	−**0.01**	(−0.03, −0.001)	**0.03**
Precipitation (average precipitation 3 months before survey)	**0.001**	(0.001, 0.002)	<**0.01**	**0.001**	(0.001, 0.002)	<**0.01**
NDVI (during previous growing season)	**0.30**	(0.03, 0.57)	**0.03**	**0.33**	(0.06, 0.60)	**0.02**
Rural residence	−**0.06**	(−0.12, −0.01)	**0.03**	−**0.07**	(−0.13, −0.01)	**0.01**
Livelihood zone [agricultural]						
Agropastoral	−0.03	(−0.12, 0.05)	0.45	−0.04	(−0.13, 0.04)	0.32
Fishing	−0.09	(−0.21, 0.03)	0.12	−0.10	(−0.21, 0.02)	0.12
Pastoral	−**0.15**	(−0.27, −0.03)	**0.01**	−**0.15**	(−0.27, −0.03)	**0.01**
*Household Level*						
Toilet facility [none]						
Flush	0.05	(−0.06, 0.16)	0.40	0.05	(−0.06, 0.16)	0.39
Non-flush	**0.10**	(0.04, 0.16)	<**0.01**	**0.10**	(0.04, 0.16)	<**0.01**
Drinking water [piped]						
Well	−**0.06**	(−0.12, −0.001)	**0.04**	−**0.06**	(−0.12, −0.001)	**0.04**
Surface	−0.04	(−0.10, 0.02)	0.16	−0.04	(−0.10, 0.02)	0.19
Other	**0.13**	(0.02, 0.23)	**0.02**	**0.13**	(0.02, 0.23)	**0.02**
Finished Flooring	**0.06**	(0.01, 0.12)	**0.02**	**0.06**	(0.01, 0.12)	**0.02**
Electricity	**0.08**	(0.01, 0.15)	**0.03**	**0.07**	(0.00, 0.14)	**0.04**
*Mother Level*						
Age (years)	0.004	(−0.001, 0.01)	0.10	0.004	(−0.001, 0.01)	0.08
Education [none]						
Primary	**0.10**	(0.03, 0.17)	**<0.01**	**0.10**	(0.03, 0.17)	**<0.01**
Secondary	**0.13**	(0.05, 0.22)	**<0.01**	**0.14**	(0.06, 0.22)	**<0.01**
*Child Level*						
Age (months)	−**0.01**	(−0.01, −0.01)	**<0.01**	−**0.01**	(−0.01, −0.01)	**<0.01**
Female	−0.01	(−0.04, 0.03)	0.69	−0.01	(−0.04, 0.03)	0.67
Birth order	−**0.02**	(−0.03, −0.001)	**<0.01**	−**0.02**	(−0.03, −0.01)	**0.01**
Recent diarrhea (yes)	−**0.06**	(−0.11, −0.001)	**0.05**	**−0.06**	(−0.11, −0.001)	**0.05**
Recent fever (yes)	−**0.09**	(−0.14, −0.05)	**<0.01**	**−0.09**	(−0.13, −0.05)	**<0.01**
AIC	165,774			165,772		
N observations	13,743					
N mothers	12,110					
N clusters	1968					

Statistically significant results are indicated in bold. Comparison groups are in brackets. Models include month of birth, survey year, and region of residence as fixed effects. Mother and DHS cluster are included as random effects

**Table 4:** Climate and Conflict Continuous WHZ score regression results for children aged 24–59 months in Nigeria

Independent variables	Model 1			Model 2		
	Coefficient	Confidence Interval	p-value	Coefficient	Confidence Interval	p-value
*ADM1 Level*						
Conflict events 1–3 months prior (count)	**−0.04**	(−0.05, −0.03)	**<0.01**			
Conflict period overlaps with growing season	**−0.84**	(−1.14, −0.54)	**<0.01**			
Conflict events 4–6 months prior (count)				−**0.03**	(−0.04, −0.02)	**<0.01**
Conflict period overlaps with growing season				−0.05	(−0.20, 0.10)	0.50
*Community Level*						
Temperature (average maximum temperature 3 months before survey)	−**0.05**	(−0.08, −0.01)	**<0.01**	−**0.08**	(−0.12, −0.04)	**<0.01**
Precipitation (average precipitation 3 months before survey)	−**0.003**	(−0.005, −0.001)	**<0.01**	−**0.004**	(−0.01, −0.002)	**<0.01**
NDVI	0.82	(−0.01, 1.64)	0.05	0.71	(−0.16, 1.58)	0.11
Rural residence	0.03	(−0.11, 0.17)	0.69	0.09	(−0.06, 0.23)	0.23
Livelihood zone [agricultural]						
Pastoral	0.23	(−0.56, 1.02)	0.57	0.28	(−0.55, 1.11)	0.50
Fishing	−0.23	(−0.56, 0.10)	0.17	−0.15	(−0.49, 0.19)	0.39
Irrigated	−**0.20**	(−0.38, −0.01)	**0.04**	−0.19	(−0.39, 0.001)	0.05
*Household Level*						
Toilet facility [none]						
Flush	0.13	(−0.04, 0.31)	0.13	0.13	(−0.04, 0.30)	0.14
Non-flush	0.06	(−0.04, 0.15)	0.25	0.06	(−0.04, 0.15)	0.22
Drinking water [piped]						
Well	−0.03	(−0.16, 0.09)	0.59	−0.05	(−0.17, 0.07)	0.44
Surface	−0.11	(−0.26, 0.05)	0.18	−0.11	(−0.27, 0.04)	0.16
Other	0.01	(−0.19, 0.22)	0.89	0.01	(−0.19, 0.22)	0.89
Finished Flooring	−0.04	(−0.12, 0.04)	0.32	−0.05	(−0.12, 0.03)	0.25
Electricity	0.08	(−0.02, 0.18)	0.11	0.08	(−0.02, 0.18)	0.11
*Mother Level*						
Age (years)	0.004	(−0.003, 0.01)	0.26	0.004	(−0.003, 0.01)	0.30
Education [none]						
Primary	0.09	(−0.004, 0.18)	0.05	0.08	(−0.01, 0.17)	0.07
Secondary	−0.003	(−0.11, 0.11)	0.96	−0.01	(−0.12, 0.10)	0.86
*Child Level*						
Age (months)	**0.004**	(0.001, 0.01)	**<0.01**	**0.004**	(0.001, 0.01)	**<0.01**
Female	**0.14**	(0.09, 0.20)	**<0.01**	**0.14**	(0.09, 0.20)	**<0.01**
Birth order	−0.01	(−0.03, 0.01)	0.34	−0.01	(−0.03, 0.01)	0.37
Recent diarrhea (yes)	−0.02	(−0.12, 0.07)	0.61	−0.02	(−0.29, −0.12)	0.66
Recent fever (yes)	−**0.21**	(−0.30, −0.12)	**<0.01**	−**0.21**	(−0.29, −0.12)	**<0.01**
AIC	164,334			164,378		
N observations	12,696					
N mothers	11,100					
N clusters	722					

Statistically significant results are indicated in bold. Comparison groups are in brackets. Models include month of birth, survey year, and region of residence as fixed effects. Mother and DHS cluster are included as random effects

**Table 5:** Climate and Conflict Continuous WHZ score regression results for children aged 24–59 months in Uganda

Independent variables	Model 1			Model 2		
	Coefficient	Confidence Interval	*p*-value	Coefficient	Confidence Interval	*p*-value
*ADM1 Level*						
Conflict events 1–3 months prior (count)	0.01	(−0.02, 0.03)	0.63			
Conflict period overlaps with growing season	−0.06	(−0.17, 0.05)	0.30			
Conflict events 4–6 months prior (count)				0.02	(−0.01, 0.05)	0.25
Conflict period overlaps with growing season				0.09	(−0.03, 0.20)	0.13
*Community Level*						
Temperature (average maximum temperature 3 months before survey)	0.02	(−0.02, 0.05)	0.36	0.02	(−0.01, 0.06)	0.14
Precipitation (average precipitation 3 months before survey)	0.0004	(−0.001, 0.001)	0.90	0.0004	(−0.0006, 0.0014)	0.42
NDVI	−0.18	(−0.69, 0.33)	0.49	0.21	(−0.71,0.30)	0.42
Livelihood zone [agricultural]						
Urban residence	0.05	(−0.12, 0.21)	0.58	0.04	(−0.12, 0.21)	0.60
Agropastoral	−0.03	(−0.13, 0.06)	0.50	−0.04	(−0.13, 0.05)	0.42
Fishing	−0.19	(−0.40, 0.01)	0.06	−0.19	(−0.40, 0.01)	0.06
Pastoral	−**0.39**	(−0.65, −0.12)	**<0.01**	−**0.38**	(−0.65, −0.12)	**<0.01**
*Household Level*						
Toilet facility [none]						
Flush	−0.10	(−0.38, 0.18)	0.48	−0.10	(−0.37, 0.18)	0.51
Non-flush	0.07	(−0.04, 0.19)	0.23	0.07	(−0.04, 0.19)	0.23
Drinking water [piped]						
Well	**0.11**	(0.00, 0.22)	**0.04**	**0.12**	(0.01, 0.23)	**0.03**
Surface	**0.15**	(0.03, 0.28)	**0.02**	**0.16**	(0.03, 0.28)	**0.01**
Other	**0.36**	(0.01, 0.71)	**0.05**	**0.35**	(0.01, 0.70)	**0.05**
Finished Flooring	0.03	(−0.06, 0.13)	0.47	0.03	(−0.06, 0.13)	0.47
Electricity	−**0.10**	(−0.21, −0.002)	**0.05**	−**0.11**	(−0.21, −0.003)	**0.04**
Mother Level						
Age (years)	**0.001**	(−0.01, 0.01)	**0.78**	0.001	(−0.01, 0.01)	**0.78**
Education [none]						
Primary	**0.05**	(−0.04, 0.14)	**0.29**	0.05	(−0.04, 0.14)	**0.26**
Secondary	**0.04**	(−0.08, 0.16)	**0.53**	0.04	(−0.08, 0.16)	**0.50**
Child Level						
Age (months)	−**0.005**	(−0.01, −0.002)	**<0.01**	−**0.005**	(−0.01, −0.002)	**<0.01**
Female	−**0.10**	(−0.16, −0.04)	**<0.01**	−**0.01**	(−0.16, −0.04)	**<0.01**
Birth order	−0.01	(−0.03, 0.02)	0.58	−0.01	(−0.03, 0.02)	0.59
Recent diarrhea (yes)	−0.03	(−0.11, 0.05)	0.46	−0.03	(−0.11, 0.05)	0.44
Recent fever (yes)	−**0.07**	(−0.13, −0.003)	**0.04**	**−0.07**	(−0.13, −0.002)	**0.04**
AIC	55,879			55,877		
N observations	4647					
N mothers	4022					
N clusters	1309					

Statistically significant results are indicated in bold. Comparison groups are in brackets. Models include month and year of birth as fixed effects. To account for correlation among siblings, mother is included as a random effect

## Footnotes

1 When a child’s WHZ is more than 3 standard deviations below the reference mean they are said to be severely wasted, when the WHZ is 2 standard deviations below the mean, they are said to be moderately wasted (UNICEF/WHO/World Bank, 2019).

2 The conflict analysis mentioned here is based on only one case: Somalia. Because of Somalia’s “fragile” status at the time of the conflict period the immediate impacts on nutrition of conflict may be specific to this unique case.

3 We explored an alternative modeling strategy using cluster adjusted standard errors (see Thiede and Strube 2020). While the results are not identical, the main results are similar and are available from the lead author on request.

4 We also combined events over the entire 6-month period (essentially merging the two different counts into a single count). However, we found this coarser temporal aggregation to be less informative and we report the results from the two models using more narrow time intervals. The 6-month results are available from the lead author.

5 We also estimate an interaction model where we include an effect for the interaction of conflict and agricultural production (NDVI) and exclude the overlapping conflict-growing season indicator variable. The interaction effect provided no additional information (in only the Kenya 4–6 month case was the effect significant). These results are presented in an abbreviated table in the “Appendix“.

## Appendix

### Description of Spatial Detail in Conflict Data

The data set codes the location of conflicts with varying degrees of spatial precision. In a share of cases events are pinpointed to particular population centers (e.g., village, town, city) or even specific neighborhoods, transportation interchanges, or physical landmarks. Other sizeable shares of cases are associated with larger geographic areas such as constituent regions of countries. For example, in the entire dataset, covering 123 countries from 1989 to 2020, 88% of the events are coded to the first-order administrative division. 42% of these events are at, or in close vicinity (25 km) of, a specific point location. Among this 42%, 23% of the points in the exact location. The countries included in our study report similar statistics.

This variation in spatial precision is due to two main reasons resulting from the data-generating process. First, certain conflict events occur with wider geographic scopes of activity, extending over areas within or across countries, rather than at point locations. The geographic coding of these events to areas is therefore factually accurate, just not spatially precise. Second, available information about events is not uniform in terms of providing complete details relevant to establishing locations with maximum spatial precision. Events may be coded with less spatial precision because the information did not permit a greater degree of spatial precision. Thus, the geographic coding of these events is accurate within the limits of the information, but not necessarily fully accurate factually.

Ultimately, the common denominator for most cases is that the user will know the locations of conflict events with certainty with respect to first-order administrative divisions at a sub-national level. Thus, this degree of spatial resolution tends to be reliable for analyses using the conflict event data where geography matters. Analysis with any more precise degrees of spatial resolution will require (1) making dubious assumptions about locations of a substantial share of cases (e.g., assigning events with imprecise spatial codings to centroids or major population centers within known areas, thereby inserting artificial precision of measurement), (2) imputing locations for those cases (thereby increasing uncertainty in measurement), or (3) dropping the cases altogether (thereby introducing incompleteness and risking bias in measurement). All of these circumstances may affect analytical results and findings (Appendix Table 6).

**Table 6:** Interacting Conflict Counts and Recent Growing Season Condition to Analyze WHZ

	Kenya Estimates (95% CD		Nigeria		Uganda	
	1–3 Months	4–6 Months	1–3 Months	4–6 Months	1–3 Months	4–6 Months
Conflict × NDVI max	0.01 (−0.03–0.05)	0.08 (0.03–0.13)	−0.10 (−0.23 −0.02)	−0.08 (−0.20–0.04)	−0.03 (−0.69–0.63)	−0.33 (−1.40–0.75)
Conflict	−0.01 (−0.03–0.01)	−**0.05 (**−**0.08–0.03)**	0.02 (−0.06–0.09)	0.02 (−0.05–0.09)	0.03 (−0.48–0.54)	0.27 (−0.58–1.12)
NDVI	0.28 (−0.04–0.61)	0.05 (−0.26–0.36)	0.93 (0.05–1.81)	0.92 (0.03–1.81)	−0.20 (−0.71–0.31)	−0.19 (−0.70–0.31)

All independent variables found in Tables 3, 4, 5 are included in these models. We present the main independent variables of interest in this abbreviated tabl

## References

[R1] Adano WR, Dietz T, Witsenburg K, & Zaal F (2012). Climate change, violent conflict and local institutions in Kenya’s drylands. Journal of Peace Research, 49(1), 65–80.

[R2] Akombi BJ, Agho KE, Renzaho AM, Hall JJ, & Merom DR (2019). Trends in socio-economic inequalities in child undernutrition: Evidence from Nigeria demographic and health survey (2003–2013). PLoS ONE, 14(2), e0211883. 30730946 10.1371/journal.pone.0211883 PMC6366715

[R3] Akresh R, Lucchetti L, & Thirumurthy H (2012). Wars and child health: Evidence from the Eritrean-Ethiopian conflict. Journal of Development Economics, 99(2), 330–340. 22962514 10.1016/j.jdeveco.2012.04.001 PMC3433062

[R4] Akresh R, Verwimp P, & Bundervoet T (2011). Civil war, crop failure, and child stunting in Rwanda. Economic Development and Cultural Change, 59(4), 777–810.

[R5] Amegbor PM, Zhang Z, Dalgaard R, & Sabel CE (2020). Multilevel and spatial analyses of childhood malnutrition in Uganda: Examining individual and contextual factors. Scientific Reports, 10(1), 1–15. 33208763 10.1038/s41598-020-76856-y PMC7676238

[R6] Arora-Jonsson S (2011). Virtue and vulnerability: Discourses on women, gender and climate change. Global Environmental Change, 21(2), 744–751.

[R7] Baker RE, & Anttila-Hughes J (2020). Characterizing the contribution of high temperatures to child undernourishment in Sub-Saharan Africa. Scientific Reports, 10(1), 1–10. 33139856 10.1038/s41598-020-74942-9 PMC7606522

[R8] Bakhtsiyarava M, Grace K, & Nawrotzki RJ (2018). Climate, birth weight, and agricultural live-lihoods in Kenya and Mali. American Journal of Public Health, 108(S2), S144–S150. 29072943 10.2105/AJPH.2017.304128 PMC5922197

[R9] Betancourt TS, & Khan KT (2008). The mental health of children affected by armed conflict: Protective processes and pathways to resilience. International Review of Psychiatry (Abingdon, England), 20(3), 317–328. 10.1080/09540260802090363 18569183 PMC2613765

[R10] Bhutta ZA, Berkley JA, Bandsma RH, Kerac M, Trehan I, & Briend A (2017). Severe childhood malnutrition. Nature Reviews Disease Primers, 3(1), 1–18. 10.1038/nrdp.2017.67 PMC7004825 28933421

[R11] Brown ME, Backer D, Billing T, White P, Grace K, Doocy S, & Huth P (2020). Empirical studies of factors associated with child malnutrition: Highlighting the evidence about climate and conflict shocks. Food Security, 1–12.

[R12] Brown ME, & de Beurs KM (2008). Evaluation of multi-sensor semi-arid crop season parameters based on NDVI and rainfall. Remote Sensing of Environment, 112(5), 2261–2271.

[R13] Buhaug H (2015). Climate–conflict research: Some reflections on the way forward. Wiley Interdisciplinary Reviews: Climate Change, 6(3), 269–275.

[R14] Bundervoet T, Verwimp P, & Akresh R (2009). Health and civil war in rural Burundi. Journal of Human Resources, 44(2), 536–563.

[R15] Corley AG (2021). Linking armed conflict to malnutrition during pregnancy, breastfeeding, and childhood. Global Food Security, 29, 100531. 33884257 10.1016/j.gfs.2021.100531 PMC8054973

[R16] Cooper MW, Brown ME, Hochrainer-Stigler S, Pflug G, McCallum I, Fritz S, (2019). Mapping the effects of drought on child stunting. Proceedings of the National Academy of Sciences, 116(35), 17219–17224. 10.1073/pnas.1905228116 PMC6717288 31405971

[R17] Currie J, & Schwandt H (2013). Within-mother analysis of seasonal patterns in health at birth. Proceedings of the National Academy of Sciences, 110(30), 12265–12270. 10.1073/pnas.1307582110 PMC3725083 23836632

[R18] Davenport F, Dorélien A, & Grace K (2020). Investigating the linkages between pregnancy outcomes and climate in sub-Saharan Africa. Population and Environment, 1–25.32836608

[R19] Davenport F, Grace K, Funk C, & Shukla S (2017). Child health outcomes in sub-Saharan Africa: A comparison of changes in climate and socio-economic factors. Global Environmental Change, 46, 72–87.

[R20] Delbiso TD, Rodriguez-Llanes JM, Donneau AF, Speybroeck N and Guha-Sapir D, (2017). Drought, conflict and children’s undernutrition in Ethiopia 2000–2013: A meta-analysis. Bulletin of the World Health Organization, 95(2). 10.2471/BLT.16.172700 PMC5327931 28250509

[R21] Dorff C, Gallop M, & Minhas S (2020). Networks of violence: Predicting conflict in Nigeria. The Journal of Politics, 82(2), 476–493.

[R22] Dunn G (2018). The impact of the Boko Haram insurgency in Northeast Nigeria on childhood wasting: A double-difference study. Conflict and Health, 12(1), 1–12. 29410702 10.1186/s13031-018-0136-2 PMC5782364

[R23] Eklund L, Degerald M, Brandt M, Prishchepov AV, & Pilesjö P (2017). How conflict affects land use: Agricultural activity in areas seized by the Islamic State. Environmental Research Letters, 12, 54004.

[R24] Funk C, Peterson P, Landsfeld M, Pedreros D, Verdin J, Shukla S, (2015). The climate hazards infrared precipitation with stations—a new environmental record for monitoring extremes. Scientific Data, 2(1), 1–21. 10.1038/sdata.2015.66 PMC4672685 26646728

[R25] Funk C, Peterson P, Peterson S, Shukla S, Davenport F, Michaelsen J, (2019). A high-resolution 1983–2016 T max climate data record based on infrared temperatures and stations by the Climate Hazard Center. Journal of Climate, 32(17), 5639–5658.

[R26] Gates S, Hegre H, Nygård HM, & Strand H (2012). Development consequences of armed conflict. World Development, 40(9), 1713–1722.

[R27] Gelman A, & Hill J (2007). Data analysis using regression and multilevel/hierarchical models (Vol. 1). Cambridge University Press.

[R28] George J, Adelaja A, & Weatherspoon D (2020). Armed conflicts and food insecurity: Evidence from Boko Haram’s attacks. American Journal of Agricultural Economics, 102(1), 114–131.

[R29] Ghobarah HA, Huth P, & Russett B (2004). The post-war public health effects of civil conflict. Social Science and Medicine, 59, 869–884. 15177842 10.1016/j.socscimed.2003.11.043

[R30] Grace K, Davenport F, Funk C, & Lerner AM (2012). Child malnutrition and climate in Sub-Saharan Africa: An analysis of recent trends in Kenya. Applied Geography, 35(1–2), 405–413.

[R31] Grace K, Davenport F, Hanson H, Funk C, & Shukla S (2015). Linking climate change and health outcomes: Examining the relationship between temperature, precipitation and birth weight in Africa. Global Environmental Change, 35, 125–137.

[R32] Grace K, Verdin A, Dorélien A, Davenport F, Funk C, & Husak G (2021). Exploring strategies for investigating the mechanisms linking climate and individual-level child health outcomes: An analysis of birth weight in Mali. Demography, 58(2), 499–526. 33834220 10.1215/00703370-8977484 PMC8382135

[R33] Hall A, Blankson B, & Shoham J (2011). The impact and effectiveness of emergency nutrition and nutrition-related interventions: A review of published evidence 2004–2010. Published Online First.

[R34] Hallegatte S, & Rozenberg J (2017). Climate change through a poverty lens. Nature Climate Change, 7(4), 250–256.

[R35] Harris J, & Nisbett N (2020). The basic determinants of malnutrition: Resources, structures, ideas and power. International Journal of Health Policy and Management. 10.34172/ijhpm.2020.259 PMC9309972 33590741

[R36] Hill R, Skoufias E, Maher B (2019). The chronology of a disaster: a review and assessment of the value of acting early on household welfare. World Bank Washington, DC. © World Bank. https://openknowledge.worldbank.org/handle/10986/31721 License: CC BY 3.0 IGO.

[R37] Högbladh S (2019). UCDP GED Codebook version 19.1, Department of Peace and Conflict Research, Uppsala University

[R38] Howell E, Waidmann T, Holla N, Birdsall N & Jiang K (2018). The impact of civil conflict on child malnutrition and mortality, Nigeria, 2002–2013. Center for Global Development Working Paper, (494).

[R39] Jia P, Lakerveld J, Wu J, Stein A, Root ED, Sabel CE, & James P (2019). Top 10 research priorities in spatial lifecourse epidemiology. Environmental Health Perspectives, 127(7), 074501. 31271296 10.1289/EHP4868 PMC6791465

[R40] Justino P (2011). The impact of armed civil conflict on household welfare and policy. IDS Working Papers, 384, 1–38.

[R41] Kandala NB, Madungu TP, Emina JB, Nzita KP, & Cappuccio FP (2011). Malnutrition among children under the age of five in the Democratic Republic of Congo (DRC): Does geographic location matter? BMC Public Health, 11(1), 261. 21518428 10.1186/1471-2458-11-261 PMC3111378

[R42] Kandel M (2016). Struggling over land in post-conflict Uganda. African Affairs, 115(459), 274–295.

[R43] Kinyoki DK, Moloney GM, Uthman OA, Kandala NB, Odundo EO, Noor AM, & Berkley JA (2017). Conflict in Somalia: Impact on child undernutrition. BMJ Global Health, 2(2), e000262. 10.1136/bmjgh-2016-000262 PMC5621625 28966793

[R44] Koren O (2018). Food abundance and violent conflict in Africa. American Journal of Agricultural Economics, 100, 981–1006.

[R45] Mansour H, & Rees DI (2012). Armed conflict and birth weight: Evidence from the al-Aqsa Intifada. Journal of Development Economics, 99(1), 190–199.

[R46] Martin-Shields CP, & Stojetz W (2019). Food security and conflict: Empirical challenges and future opportunities for research and policy making on food security and conflict. World Development, 119, 150–164.

[R47] Minoiu C, & Shemyakina ON (2014). Armed conflict, household victimization, and child health in Côte d’Ivoire. Journal of Development Economics, 108, 237–255.

[R48] Niles MT, & Brown ME (2017). A multi-country assessment of factors related to smallholder food security in varying rainfall conditions. Scientific Reports, 7(1), 1–11. 29176578 10.1038/s41598-017-16282-9 PMC5701123

[R49] Perez-Heydrich C, Warren JL, Burgert CR, & Emch ME (2016). Influence of demographic and health survey point displacements on raster-based analyses. Spatial Demography, 4(2), 135–153. 29888316 10.1007/s40980-015-0013-1 PMC5993452

[R50] Petersen LK (2018). Real-time prediction of crop yields from MODIS relative vegetation health: A continent-wide analysis of Africa. Remote Sensing, 10(11), 1726.

[R51] Phalkey RK, Aranda-Jan C, Marx S, Höfle B, & Sauerborn R (2015). Systematic review of current efforts to quantify the impacts of climate change on undernutrition. Proceedings of the National Academy of Sciences, 112(33), E4522–E4529. 10.1073/pnas.1409769112 PMC4547305 26216952

[R52] Prado EL, Jimenez EY, Vosti S, Stewart R, Stewart CP, Somé J, & Dewey K (2019). Path analyses of risk factors for linear growth faltering in four prospective cohorts of young children in Ghana, Malawi and Burkina Faso. BMJ Global Health, 4(1), e001155. 10.1136/bmjgh-2018-001155 PMC6350712 30775005

[R53] Randell H, Grace K, & Bakhtsiyarava M (2021). Climatic conditions and infant care: Implications for child nutrition in rural Ethiopia. Population and Environment, 42(4), 524–552. 34149138 10.1007/s11111-020-00373-3 PMC8210853

[R54] Randell H, Gray C, & Grace K (2020). Stunted from the start: Early life weather conditions and child undernutrition in Ethiopia. Social Science & Medicine, 261, 113234. 32823214 10.1016/j.socscimed.2020.113234 PMC7716344

[R55] Rowhani P, Degomme O, Guha-Sapir D, & Lambin EF (2012). Malnutrition and conflict in Eastern Africa: Impacts of resource variability on human security. In Scheffran J, Brzoska M, Brauch HG, Link PM, & Schilling J (Eds.), Climate change, human security and violent conflict (pp. 559–571). Springer.

[R56] Shukla S, Husak G, Turner W, Davenport F, Funk C, Harrison L, (2021). A slow rainy season onset is a reliable harbinger of drought in most food insecure regions in Sub-Saharan Africa. PLoS ONE, 16(1), e0242883. 33471787 10.1371/journal.pone.0242883 PMC7816988

[R57] Sundberg R, & Melander E (2013). Introducing the UCDP georeferenced event dataset. Journal of Peace Research, 50(4), 523–532.

[R58] Teal RK, Tubana B, Girma K, Freeman KW, Arnall DB, Walsh O, & Raun WR (2006). In-season prediction of corn grain yield potential using normalized difference vegetation index. Agronomy Journal, 98(6), 1488–1494.

[R59] Tesfai C, Ratnayake R, & Myatt M (2013). Measuring local determinants of acute malnutrition in Chad: A case-control study. The Lancet, 381, S144.

[R60] Theisen OM (2012). Climate clashes? Weather variability, land pressure, and organized violence in Kenya, 1989–2004. Journal of Peace Research, 49(1), 81–96.

[R61] Thiede BC, Hancock M, Kodouda A, & Piazza J (2020). Exposure to armed conflict and fertility in Sub-Saharan Africa. Demography, 57(6), 2113–2141. 33067758 10.1007/s13524-020-00923-2 PMC8141372

[R62] Thiede BC, & Strube J (2020). Climate variability and child nutrition: Findings from sub-Saharan Africa. Global Environmental Change, 65, 102192. 34789965 10.1016/j.gloenvcha.2020.102192 PMC8594912

[R63] Tuholske C, Caylor K, Funk C, Verdin A, Sweeney S, Grace K, (2021). Global urban population exposure to extreme heat. Proceedings of the National Academy of Sciences, 118(41). 10.1073/pnas.2024792118 PMC8521713 34607944

[R64] UNICEF/WHO/World Bank Joint Child Malnutrition Estimates, March 2019 edition.

[R65] van Cooten MH, Bilal SM, Gebremedhin S, & Spigt M (2019). The association between acute malnutrition and water, sanitation, and hygiene among children aged 6–59 months in rural Ethiopia. Maternal & Child Nutrition, 15(1), e12631. 29961977 10.1111/mcn.12631 PMC7232102

[R66] Verdin A, Funk C, Peterson P, Landsfeld M, Tuholske C, & Grace K (2020). Development and validation of the CHIRTS-daily quasi-global high-resolution daily temperature data set. Scientific Data, 7(1), 1–14. 32929097 10.1038/s41597-020-00643-7 PMC7490712

[R67] Verpoorten M (2009). Household coping in war-and peacetime: Cattle sales in Rwanda, 1991–2001. Journal of Development Economics, 88(1), 67–86.

[R68] Vrieling A, de Beurs KM, & Brown ME (2008, October). Recent trends in agricultural production of Africa based on AVHRR NDVI time series. In Neale CMU, Owe M, & D’Urso G (Eds.), Remote sensing for agriculture, ecosystems, and hydrology X (Vol. 7104, p. 71040R). International Society for Optics and Photonics.

[R69] WHO (2017). Diarrhoeal disease. https://www.who.int/news-room/fact-sheets/detail/diarrhoeal-disease. Accessed 9 October 2020.

[R70] WHO (2020). Children: Improving Survial and Wellbeing. https://www.who.int/news-room/fact-sheets/detail/children-reducing-mortality. Accessed 3 November 2021.

